# Inhibition of fibrosis and inflammation by triple therapy with pirfenidone, edaravone and erythropoietin in rabbits with drug-induced lung injury: comparison of CT imaging and pathological findings

**DOI:** 10.3892/etm.2013.1308

**Published:** 2013-09-18

**Authors:** SHOBU WATANABE, NORIHISA NITTA, AKINAGA SONODA, AYUMI NITTA-SEKO, SHINICHI OHTA, KEIKO TSUCHIYA, HIDEJI OTANI, YUKI TOMOZAWA, YUKIHIRO NAGATANI, KENICHI MUKAISHO, MASASHI TAKAHASHI, KIYOSHI MURATA

**Affiliations:** Department of Radiology, Shiga University of Medical Science, Otsu, Shiga 520-2192, Japan

**Keywords:** bleomycin, anticancer drug, reactive oxygen species, monotherapy, combined administration

## Abstract

In a rabbit model of bleomycin-induced lung injury, computed tomography (CT) and pathological studies were conducted to investigate whether the progression of this injury is inhibited by pirfenidone and by triple therapy with pirfenidone, edaravone and erythropoietin. We divided nine rabbits with bleomycin-induced lung injury into three equally sized groups. Group 1 served as the control, group 2 received pirfenidone alone and group 3 was treated with pirfenidone, edaravone and erythropoietin. Multidetector CT (MDCT) scans were acquired immediately after the administration of bleomycin, and further scans were performed on days 14 and 28. The area of abnormal opacity was calculated. The rabbit lungs were removed and the size of abnormal areas in macroscopic specimens was calculated and the degree of fibrosis and inflammation in microscopic specimens was scored. In order, the average size of the area of abnormal opacity on CT scans was largest in group 1, followed by groups 2 and 3. On day 28, the area of opacity was significantly smaller in group 3 than in group 1 (P=0.071). The average size of the area of abnormal opacity on macroscopic findings was largest in group 1, followed in order by groups 2 and 3; the difference between group 1 and 3 was significant (P<0.05). The average fibrosis score was highest in group 3 followed by groups 2 and 1. By contrast, the average inflammation score was highest in group 2 followed by groups 1 and 3. Although the administration of pirfenidone alone slowed the progression of bleomycin-induced lung injury, the triple-drug combination was more effective.

## Introduction

A number of drugs may produce lung injury and novel anticancer drugs, including molecular targeted drugs, are causative of severe lung injury (1–3). The development of novel anticancer drugs and biological agents may result in an increase in the incidence of drug-induced lung injury.

Although poorly understood, direct cellular injury by anti-cancer drugs and their metabolites, and/or indirect injury via the activation of immune cells have been suggested as mechanisms underlying the onset of drug-induced lung injury (4,5). The onset of lung injury is also modified by a variety of background factors, including genes involved in drug metabolism, genetic factors, such as immunity-related genes, patient age, total drug dose, synergistic effects of drug combinations, previous or current radiation therapy, background respiratory disease, a history of smoking, oxygen inhalation/respirator management, bone marrow/stem cell transplants and renal failure (6,7).

At present there is no appropriate non-invasive clinical test for drug-induced lung injury. Pathological and imaging findings are non-specific and do not facilitate the identification of causative drugs (8). In addition, the same drug may produce different patterns of lung injury depending on the dose and individual responses. While steroids and immunosuppressants are used to suppress lung inflammation, they fail to improve the prognosis of patients (9).

The fibrosis inhibitor pirfenidone (10), the antioxidant edaravone (11) and the cytoprotective agent erythropoietin (12) have different mechanisms of action. Using a rabbit model of bleomycin-induced lung injury we studied the effectiveness of these drugs in suppressing lung tissue injury. We evaluated time-course changes on computed tomography (CT) images and compared concurrent imaging and pathological findings.

## Materials and methods

This study was approved by the Ethics Committee on Animal Experiments of Shiga University of Medical Science (Otsu, Japan).

### Animal model and drugs

Nine Japanese white rabbits (SLC Inc., Shizuoka, Japan) weighing 3 kg were divided into three equal groups. Group 1 served as the control, group 2 received monotherapy with pirfenidone (Shionogi & Co. Ltd., Osaka, Japan) and group 3 was treated with pirfenidone, edaravone (Mitsubishi Pharma Co., Osaka, Japan), and erythropoietin (Chugai Pharma Co., Ltd., Tokyo, Japan). Bleomycin hydrochloride (30 mg; Nihon Kayaku Co., Tokyo, Japan), dissolved in 2 ml physiological saline, was delivered into the trachea by a 22-gauge indwelling needle. To obtain drug distribution throughout the lungs, 0.5 ml of this solution was administered iteratively with postural changes of the recipients. Pirfenidone was administered orally (90 mg/day) for 28 days. Edaravone and erythropoietin were intravenously administered at 3.6 mg/day for 7 days and 30,000 IU/day for 3 days, respectively.

### CT examination

CT images were acquired on a four-row multidetector computed tomography (MDCT) scanner (Toshiba Medical Systems Corporation, Tokyo, Japan) before, immediately after and 14 and 28 days after the administration of bleomycin. The scanning parameters were as follows: X-ray tube voltage, 120 kV; X-ray tube current, 50 mA; collimation, 1 mm; field of view, 100 mm; and helical pitch (HP), 0.8. Following CT scanning on day 28, the rabbits were euthanized and their lungs were resected. Inflated fixed lungs were prepared according to the method of Heitzman (13).

The CT images were reconstructed; horizontal 1-mm thick cross-sections were constructed at 3-mm intervals from the apex of the lungs in the lung window. The images were recorded using Aquarius NetStation iNtuition Edition (Tera Recon Inc., San Mateo, CA, USA) and analyzed with Segmentation Analysis and Tracking (SAT) image software (Tera Recon Inc., San Mateo, CA, USA). Two radiologists individually measured the normal and affected areas (ground-glass opacities and infiltrative shadows) in each cross-section, calculated the average area of abnormal shadows in the lung field and evaluated time-course changes. When the measurements were consistent they were adopted for analysis.

### Histopathological examination

The resected lungs were fixed in formaldehyde and consecutive 4-*μ*m thick slices were stained with hematoxylin and eosin and Elastica van Gieson stain. One cross-section on each glass slide was selected from the center of the craniocaudal axis in the bilateral anterior and posterior lobes. These slides were scanned with Coolscan (Nikon, Tokyo, Japan) for macroscopic evaluation. Lesions were measured using ImageJ software (version 1.43, US National Institutes of Health) and the average size was compared among the three groups. Macroscopic images (magnification, ×200) were evaluated by one pathologist and one radiologist specializing in chest diseases. The evaluators consensually scored the degree of inflammatory cell infiltration in the alveolar wall and alveoli using the method of Hirose *et al* (14), on a scale of 0 (mild) to 5 (severe). The sum of the scores (0–10) in the bilateral anterior and posterior lobes of each rabbit was recorded and the average was calculated to evaluate inflammatory changes. To evaluate the degree of fibrosis, interstitial fibrosis and the formation of honeycomb lung lesions were recorded on a score of 0 to 5 and the degree of alveolar metaplasia and smooth muscle proliferation in interstitial tissue was recorded on a score of 0 to 2. The average of the sum of the scores, from 0 to 14, in the bilateral anterior and posterior lobes was evaluated (14). Microscopic slides were examined by a pathologist with 16 years of experience.

### Statistical analysis

CT and pathological findings from the three groups of rabbits were compared using the t-test. Findings from microscopic images were assessed using Tukey’s honestly significant difference (HSD) test. P<0.05 was considered to indicate a statistically significant difference; a trend toward a significant difference was considered to be present at 0.05<P<0.10.

## Results

### CT examination

**Figure 1.**
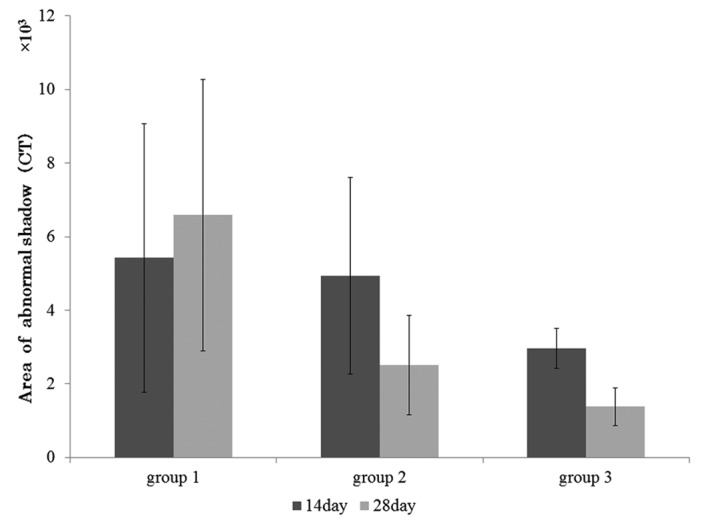
Effect of different treatments on the abnormal area, as measured using computed tomography (CT). The area of abnormal shadow in group 1 was the largest of the three groups and the area of abnormal shadow in group 3 was the smallest of the three groups. On day 28, the area of abnormal shadow in group 1 was markedly higher compared with that in group 3 (P=0.071). Group 1, control; group 2, monotherapy with pirfenidone; group 3, triple therapy with pirfenidone, edaravone and erythropoietin.

On CT images acquired 14 and 28 days after the administration of bleomycin, the average size of the abnormal areas was largest in the control group, followed by rabbits subjected to monotherapy and triple therapy (groups 1, 2 and 3, respectively; Fig. 1 and Table I). The mean values for groups 1, 2 and 3 on day 14 were 5.42, 4.95 and 2.96×10^3^ mm^2^, respectively and on day 28 were 6.59, 2.51 and 1.38×10^3^ mm^2^, respectively. While there was no significant difference among the three groups on day 14, on day 28 the abnormal area was markedly smaller in group 3 compared with that in group 1 (P=0.071). There was no significant difference between groups 2 and 3. On day 28 the area with abnormal shadows was smaller compared with that on day 14 in the two experimental groups, while it was slightly larger in the controls.

### Histopathological examination

#### Macroscopic findings.

**Figure 2.**
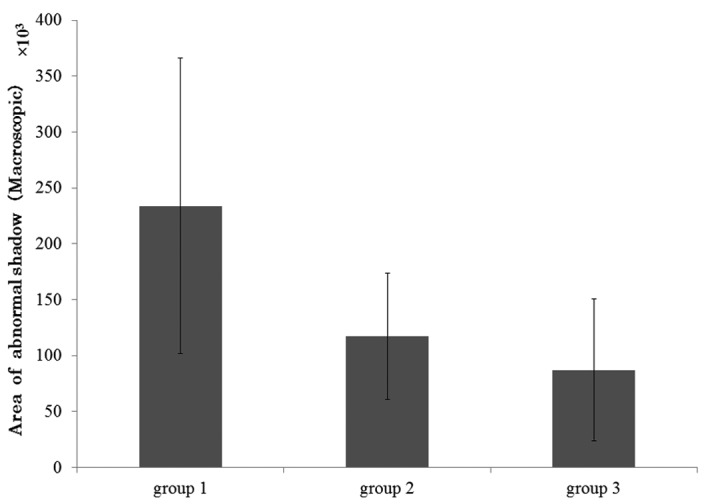
Size of abnormal area in macroscopic specimens. The largest macroscopic abnormal area in the pathological specimens was in group 1, followed by groups 2 and 3. There was a significant difference between groups 1 and 3 (P<0.05) and a marked difference between groups 1 and 2 (P= 0.09). Moreover, there was no significant difference between groups 2 and 3. Group 1, control; group 2, monotherapy with pirfenidone; group 3, triple therapy with pirfenidone, edaravone and erythropoietin.

Macroscopically, group 1 exhibited the largest abnormal areas; average size of abnormal areas, 233.80×10^3^ (group 1) vs. 117.25×10^3^ (group 2) and 87.1×10^3^ *μ*m^2^ (group 3). The difference between groups 1 and 3 was significant (P<0.05) and there was a marked difference (P= 0.09) between groups 1 and 2. There was no significant difference between groups 2 and 3 (Fig. 2 and Table II).

#### Microscopic findings.

**Figure 3.**
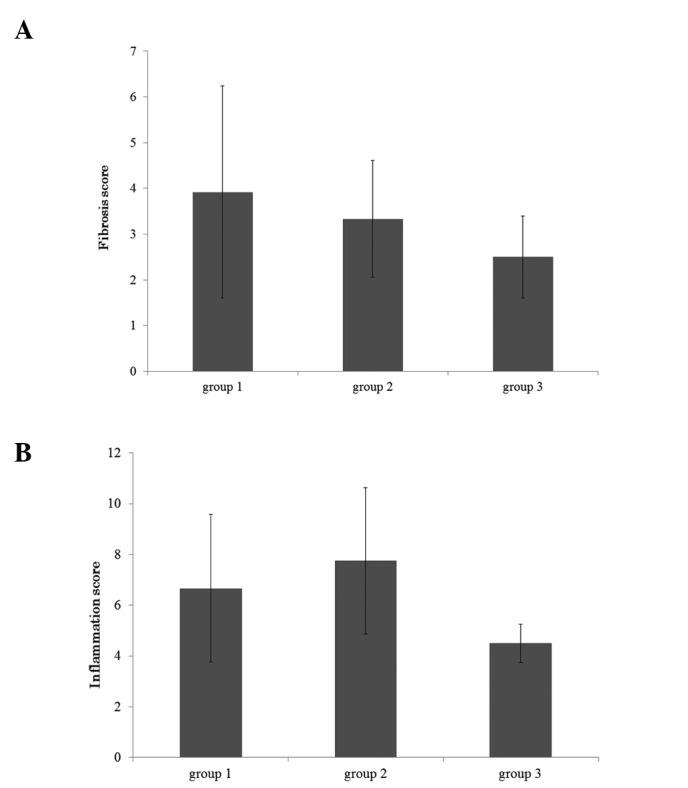
Microscopic findings. (A) Fibrosis score; (B) Inflammation score. Microscopic findings in pathological specimens revealed the average fibrosis score was the highest in group 1, followed by groups 2 and 3. The highest average inflammation score was in group 2, followed by groups 1 and 3. There were no significant differences among the three groups. Group 1, control; group 2, monotherapy with pirfenidone; group 3, triple therapy with pirfenidone, edaravone and erythropoietin.

The average fibrosis score was highest in group 1 followed by groups 2 and 3 (group 1, 3.92; group 2, 3.33; group 3, 2.50; Fig. 3A and Table III). Group 2 presented the highest average inflammation score, followed by groups 1 and 3 (control, 6.67; group 2, 7.75; group 3, 4.50; Fig. 3B and Table III). There was no significant difference in the average fibrosis score among the three groups.

## Discussion

Among anticancer drugs, bleomycin elicits the highest rate of drug-induced interstitial pneumonitis and pulmonary fibrosis; ∼10% of patients treated with bleomycin develop lung injuries (15). Molecules of this drug harbor an iron-binding site and an iron ion induces a free radical, which in turn plays a role in cleaving DNA.

Although the mechanism(s) by which bleomycin induces pulmonary fibrosis remain to be elucidated, reactive oxygen species (ROS) produced by bleomycin directly injure the lung epithelium and endothelium (16–18). Early lung injuries induce an increase in the recruitment of activated inflammatory cells into the lung parenchyma. The production of ROS by inflammatory cells, including alveolar macrophages and polymorphonuclear leukocytes, is considered to be closely associated with the pathogenesis of bleomycin-induced pulmonary fibrosis (19).

Fibrosis inhibitors, anti-oxidants and cytoprotective agents have been evaluated for ability to attenuate the effects of bleomycin. Pirfenidone, a fibrosis inhibitor, was first approved in 2008 for the treatment of idiopathic pulmonary fibrosis (20). The production of inflammatory cytokines, including tumor necrosis factor (TNF)-α, interleukin (IL)-1 and IL-6 is suppressed while the production of anti-inflammatory cytokines, including IL-10 is stimulated by this drug. These activities suppress the reduction in the interferon (IFN)-γ level and this corrects the imbalance in Th2-type predominant reactions (i.e., correction of the Th1/Th2 balance). As pirfenidone also suppresses the production of growth factors related to fibrosis formation, including transforming growth factor (TGF)-β1, basic fibroblast growth factor (b-FGF) and platelet-derived growth factor (PDGF), it regulates the production of a variety of cytokines and growth factors (21,22). The suppressive effect of pirfenidone on fibroblast proliferation and collagen production may be involved in its suppression of fibrosis.

The antioxidant edaravone, approved for use in Japan in April 2001, is the first brain protective agent (23). It eliminates or detoxifies harmful free radicals and protects the brain from oxidative stress. It has been reported that the inhibition of interstitial edema and of infiltration by inflammatory cells via ROS suppresses the progress of pulmonary fibrosis (18).

Erythropoietin, a hematopoietic cytokine produced in the kidneys, induces erythroblast differentiation and plays a major role in stimulating hematopoiesis. It also stimulates apoptosis-inhibitory factors, inhibits apoptosis by inhibiting apoptosis-inducing factors, and exerts cytoprotective activity by binding the erythropoietin receptor (EPOR) expressed in the heart, vascular system and brain. As human recombinant erythropoietin (rhEPO) directly inhibits pulmonary fibrosis by binding to EPOR on alveolar and bronchial epithelial cells (24), it may promote the healing of these cells damaged by anticancer drugs via the induction of vascular endothelial precursor cells in the bone marrow, thereby indirectly inhibiting pulmonary fibrosis (25).

In the present study we compared abnormal shadows on CT scans in rabbits. The results demonstrate that the area of abnormal shadows was slightly larger on day 28 compared with that on day 14 after bleomycin administration, which may be attributable to persistent, aggravated drug-induced lung injury. At the two time-points, these areas were smallest in the triple therapy group; in the two experimental groups, the average size of abnormal areas was significantly smaller on day 28 compared with day 14. This observation suggests that the combined administration of edaravone, erythropoietin, and pirfenidone more potently suppressed the progression of drug-induced lung injury than monotherapy with pirfenidone.

While we identified no difference among the three groups on day 14 after bleomycin administration, on day 28 there was a marked difference between the controls and rabbits subjected to triple therapy. According to Nagatani *et al* (26) and Izbicki *et al* (27), the tracheal delivery of bleomycin results in lung injury, which suggests that interstitial inflammation and edematous changes occur in the early stage prior to the manifestation of fibrotic changes. We posit that the abnormal shadows we detected on CT scans obtained on day 14 were indicative of not only early-stage fibrosis, but also of inflammatory changes, and that images acquired on day 28 demonstrated fibrotic changes following the amelioration of interstitial inflammation. Comparison of groups 2 and 3 revealed that triple therapy resulted in the prolonged, potent suppression of fibrosis in rabbits treated with bleomycin.

Fibrotic and inflammatory changes were compared in the present study. Macroscopically, the average area of abnormal shadows was largest in group 1 followed, in order, by groups 2 and 3. There was a marked difference between the controls and group 1 (P=0.09) and there was a significant difference between the controls and group 3 (P<0.05). These findings suggest that bleomycin-induced lung injury, including inflammation and fibrosis of lung tissue, was reduced by monotherapy with pirfenidone and by triple therapy with edaravone, erythropoietin and pirfenidone.

Microscopic analysis revealed no significant difference in the fibrosis score of the three groups; however, the severity of fibrosis was greater in group 1 compared with that in groups 2 and 3, and greater in group 2 than in group 3, suggesting that triple therapy suppressed fibrosis more potently than monotherapy.

Although there was no significant difference in the inflammation score among the three groups, the average score for inflammatory changes was the highest in group 2, followed by group 1 and group 3. We offer two explanations for the difference between this observation and our CT and pathological findings: i) Fibrotic changes are the final stage while inflammatory changes are the mid-term process of drug-induced lung injury (26,27). In the rabbits in the present study, lung inflammation had subsided and the development of fibrosis had begun by day 28 after bleomycin administration. The observation that inflammation subsided earlier in the control than in the monotherapy group supports this hypothesis; ii) since monotherapy with pirfenidone suppresses the production of inflammatory cytokines and stimulates the production of anti-inflammatory cytokines, inflammatory changes may have advanced more slowly in rabbits treated with pirfenidone than in the controls where these changes quickly shifted to fibrosis.

As we acquired all the pathological samples on day 28 after the delivery of bleomycin, we were unable to perform time-course studies of the lung injury induced by bleomycin.

We attribute the absence of significant differences in the fibrosis and inflammation scores among the three groups to our selection process for microscopic evaluation, since only one slide from bilateral specimens of the anterior and posterior lobes was used. Consequently, the slides we studied may not have included portions with severe fibrosis and inflammatory changes. In future studies we will refer to CT images to identify areas exhibiting severe changes prior to selecting the slides to be used for pathological evaluation.

The mechanism(s) underlying the lung injury induced by drugs remain poorly understood. With respect to anticancer drugs and their metabolites, indirect mechanisms based on allergic reactions have been suggested (28), although both direct and indirect mechanisms may result in drug-induced lung injury.

Direct injury to lung capillary and alveolar epithelial cells results in increased vascular permeability and interstitial edema, and promotes fibrosis by stimulating fibroblasts. As these changes are drug dose-dependent and become chronic, the onset of drug-induced lung injury may be predictable. Indirect cellular injury as a consequence of allergic reactions is induced by drugs and their metabolites; changes in immune reactions evoked by drugs and the involvement of type III and IV reactions are suspected (29–32). As these changes are not dose-dependent, it is not possible to predict their manifestation and in certain instances an acute clinical course may lead to respiratory insufficiency within a few days of the start of drug administration (33). In addition, depending on the patient’s background, a variety of clinical courses and symptoms may be elicited in patients receiving the same drug. This suggests the involvement of different mechanisms in the elicitation of drug-induced lung injury.

The rabbit model of bleomycin-induced lung injury used in the present study demonstrated that triple therapy with pirfenidone, edaravone and erythropoietin was more effective than monotherapy with pirfenidone. We posit that triple therapy suppressed lung fibrosis more effectively than monotherapy since the different mechanisms of action of pirfenidone, edaravone and erythropoietin concurrently slowed the progression of lung fibrosis.

Our study has a number of limitations. The distribution of bleomycin in the lungs may not have been uniform among individual rabbits and injury in different lung areas may have resulted in differences in the degree and distribution of abnormal areas among the animals. To assess the degree of suppression of lung fibrosis by the administered drugs more accurately, studies are underway to determine the rate change in abnormal areas.

In conclusion, since allergy-based lung injury induced by anticancer drugs progresses quickly and leads to respiratory insufficiency within days, immediate and appropriate treatment is imperative. We demonstrated that the combined administration of pirfenidone, edaravone and erythropoietin effectively limited the extent of lung damage in a rabbit model of bleomycin-induced lung injury.

## Figures and Tables

**Table I.** Effect of different treatments on the size of the abnormal area, as measured using CT (mm^2^).

Group	Rabbit 1	Rabbit 2	Rabbit 3
Day 14			
Group 1	5,255.63	1,849.79	9,151.77
Group 2	6,460.87	6,497.77	1,863.44
Group 3	3,546.37	2,865.62	2,484.55
Day 28			
Group 1	2,711.62	10,062.89	6,998.04
Group 2	3,276.21	3,310.37	937.38
Group 3	1,748.98	1,589.65	798.37

Group 1, control; group 2, monotherapy with pirfenidone; group 3, triple therapy with pirfenidone, edaravone and erythropoietin. CT, computed tomography.

**Table II.** Size of the abnormal area in the macroscopic pathology image of each group (*μ*m^2^).

Group	Rabbit 1	Rabbit 2	Rabbit 3
Group 1	271647.5	319624.5	110117.3
Group 2	115202.5	61994.0	128822.8
Group 3	144483.3	67646.3	49180.5

Group 1, control; group 2, monotherapy with pirfenidone; group 3, triple therapy with pirfenidone, edaravone and erythropoietin.

**Table III.** Microscopic findings (fibrosis and inflammation score).

Scores	Rabbit 1	Rabbit 2	Rabbit 3
Fibrosis score			
Group 1	7.75	2.75	1.25
Group 2	2.25	4.75	3.00
Group 3	2.75	1.50	3.25
Inflammation score			
Group 1	10.00	4.75	5.25
Group 2	4.50	8.75	10.00
Group 3	4.50	3.75	5.25

Group 1, control; group 2, monotherapy with pirfenidone; group 3, triple therapy with pirfenidone, edaravone and erythropoietin.
